# Human Attention and Visual Cognition: Introduction

**DOI:** 10.3390/jimaging8120318

**Published:** 2022-11-28

**Authors:** Thomas Sanocki

**Affiliations:** Department of Psychology, University of South Florida, Tampa, FL 33620, USA; sanocki@usf.edu

In a world that is increasingly fast and complex, the human ability to rapidly perceive, comprehend, and act on visual information is extremely important. *Visual Cognition* is the study of these processes. Additionally, given the complexity of stimulus information, prioritization and selection—processes of *attention*—are central to visual cognition. This Special Issue will present cutting edge, informative, and readable research on these topics for a wide audience of intelligent readers, in the form of general *Review* papers and interesting *Studies*.

One of the most remarkable abilities of the human brain is the ability to “grasp” the meaning of a new (unexpected) complex scene in only a fraction of a second. This achievement results from sophisticated parallel and concurrent processes that begin with the stimulus scene but rapidly integrate relevant semantic and other knowledge in the brain. The first Review paper, by Nurit Gronau [1], presents an incisive program of research on the role of attention as vision meets semantic knowledge. The research uses meaningful stimuli such as objects and scenes, together with careful experimental control of spatial attention and task relevance in brief visual displays. The experiments arrive at well-articulated conclusions about attention, task, and rapid visual comprehension.

The Review paper by Thomas Sanocki and Han Lee [2] addresses top-down attention. What are the most powerful effects of intentions and goals on mental processing? The paper begins with a historical perspective that refers to the major topics in the field of attention. Then, the paper focuses on two of the most powerful experimental effects on mental processing, one involving the set-up of tasks, and one involving the setting of attention in time. The paper then extends the temporal aspect of attention, with the idea that attention sets up major processes over the time scale of seconds, with significant consequences.

Because our human subjects come fully equipped with sophisticated machinery, behavioral science has not fully appreciated the resource and computational challenges that exist. Of course, these challenges become obvious when one builds a vision machine from scratch, as computer imaging and vision researchers are aware. John Tsotsos is a scholar who thinks in both the human and machine worlds, and has studied computational and resource issues related to attention and vision throughout his distinguished career. He graciously accepted our invitation to write a perspective paper on attention [3]. Addressing computation and attention from a high vantage point, he begins by posing an essential question about the study of attention: “What exactly are we trying to understand?” Then, Tsotsos distills claims about attention and vision at David Marr’s level of Computational Theory, providing perspectives that can help define and mature the study of attention and vision.

The discovery and development of interdisciplinary relations is good for the cognitive and imaging sciences, but there are few established venues for new interdisciplinary empirical research. Thus, we are happy to present two empirical *Studies* that help bridge the computer imaging and behavioral worlds. These papers are creative and empirical, but less overwhelming than the typical behavioral paper. *Studies* are published for their novelty and interest to a wide audience, under the JOI heading *Research*. Naoyuki Awano and Yuki Hayashi [4] studied a feature computed from image contours: Psychological Field Potential. The researchers examine the potential for this feature to aid object categorization, comparing it and other image features in their ability to predict the record of human eye fixations, as humans categorize line-drawing images.

Serena Mandolesi and colleagues [5] take us out of the laboratory and into a palace (!). Specifically, they show us one room full of history and historical images—the *Studiolo del Duca* in Urbino, Italy. In this room, there is plenty to look at—but how can we study humans attending to the images as they move freely through the room? Using the technology of eye-tracking spectacles, the researchers follow and map the attention of non-expert visitors, gaining clues about patterns of comprehension.

Each paper has been carefully reviewed for integrity and for readability, along with standard criteria. We hope you will find informative and enjoyable reading.
